# Retained Foreign Body in the Breast Following a Motor Vehicle Collision: A Case Report

**DOI:** 10.1155/2024/5262600

**Published:** 2024-09-26

**Authors:** Daniela Bresciani, Jacqueline Tsai

**Affiliations:** Breast Surgical Oncology, Department of General Surgery, Stanford University School of Medicine, Stanford, California, USA

## Abstract

**Background:** Unintentionally retained foreign bodies in the breast are a rare phenomenon. Most reported cases are iatrogenically derived from surgeries and procedures. Only a handful of reported cases refer to noniatrogenic causes, including bullets, a sewing needle, and a headscarf pin. However, there are no reports to date that describe a retained foreign body in the breast after a motor vehicle collision or a similar traumatic event or from a decorative steering wheel emblem decal.

**Case Description:** We report the case of a 25-year-old female who was involved in a motor vehicle collision with airbag deployment that led to a left breast retained foreign body, a steering wheel emblem decal. On presentation to the emergency room, she reported left chest pain associated with a puncture wound lateral to the left nipple. Imaging at that time was consistent with a metallic object embedded in the subcutaneous tissue of the left breast. Four months after the accident, the patient continued having daily burning pain in the associated area. As such, surgical excision was recommended, and wire-localized excision of the foreign body was subsequently performed. Grossly, the foreign body appeared as a metallic object with rhinestones, which the patient confirmed was a decorative emblem decal that was on her steering wheel. The postoperative course was uncomplicated, and follow-up examinations revealed resolution of the left breast pain.

**Conclusions:** This case underscores a unique presentation after a common accident—a retained foreign body in the breast after a motor vehicle collision—and its successful surgical intervention leading to a favorable postoperative course. Notably, the National Highway Traffic Safety Administration recently advised drivers against adding decorative emblem decals to their steering wheels for this reason. The case therefore highlights safety precautions that should be taken regarding the addition of this type of accessory.

## 1. Introduction

Retained foreign bodies in the breast are extremely rare and comprise only 0.7% of unintentionally retained foreign objects in the body [1]. Most etiologies originate from surgeries and medical procedures, involving items such as sponges, guide wire pieces, surgical clips, and biopsy needles [2, 3]. Noniatrogenic causes are even rarer, with only a few documented cases in the literature, primarily involving retained bullets or bullet fragments [3]. Occasionally, other unconventional foreign bodies, such as a sewing needle, headscarf pin, door handle, and a piece of hair, have been reported [4–7].

In 2022 in the United States, there were an estimated 5.9 million police-reported motor vehicle traffic crashes, resulting in ~2.17 million vehicle occupants being injured [8]. Among these injuries, the deployment of airbags can occasionally lead to unusual trauma, including the introduction of foreign objects into the body [9, 10]. Additionally, decorative steering wheel emblem decals, which are ornamental pieces typically made of metal or plastic that are adhesively attached to the center of steering wheels, can be one such introduced foreign body during a collision. There has been only one very recent report of a penetrating injury caused by this type of accessory, which involved the neck and face [9].

Interestingly, despite the high incidence of motor vehicle accidents, there are no documented cases of retained foreign bodies in the breast resulting from motor vehicle accidents or involving decorative steering wheel emblem decals. Notably, breast trauma is an often underrecognized injury in female trauma patients, as reported in a recent National Trauma Data Bank review [11]. We present a unique case of a retained foreign body—a decorative steering wheel emblem decal—in the breast following a motor vehicle collision with airbag deployment.

## 2. Case Presentation

**Figure 1:**
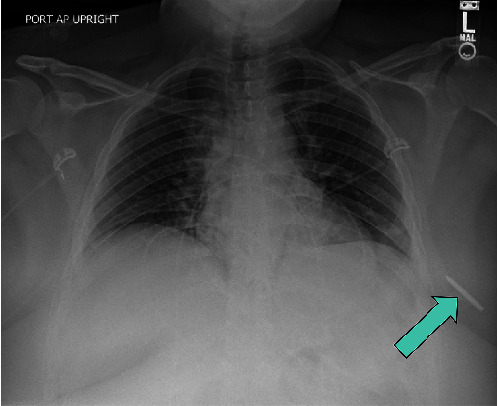
Chest X-ray demonstrating the foreign body in the left breast.

**Figure 2:**
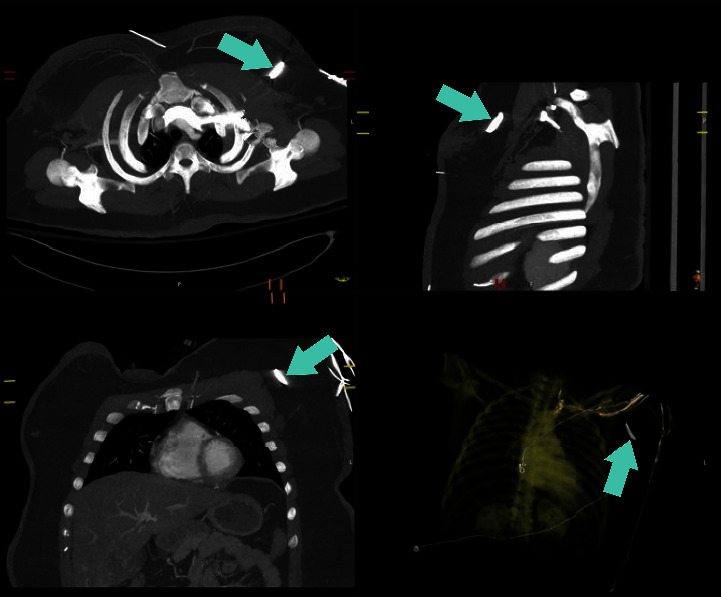
CT angiogram demonstrating the metallic foreign body.

A 25-year-old obese female presented to the emergency department complaining of constant, 8/10 left chest pain and brief loss of consciousness following a motor vehicle collision with airbag deployment. Initial assessments, including primary and secondary surveys and medical history, were unremarkable. On physical exam, her vital signs were stable, and the patient had a notable BMI of 52.1 kg/m^2^. On examination of her chest, abrasions extending from her left neck to her upper arm and anterior chest were found. On breast examination, there was a puncture wound at the 3 o'clock position just lateral to the nipple, and there were no palpable masses. A chest X-ray (Figure 1) revealed a 3.2-cm linear radiopaque foreign body in the left upper chest. This was subsequently confirmed by CT angiography of the chest (Figure 2) as a 3.2 cm × 0.6 cm metallic object embedded in the subcutaneous tissue with associated soft tissue tract and hematoma, noted to likely be debris from the accident. The patient received fentanyl at the ED for pain control. As the patient was hemodynamically stable and there were no other significant findings on exam, her superficial wounds were treated, and she was discharged home on oxycodone and a course of antibiotics.

On a follow-up phone call 2 days later, the patient denied any further chest pain. On follow-up appointment with general surgery a week after discharge, the patient described consistent pain and a burning sensation localized to the area on the breast where the fragment was previously seen on imaging. Physical examination was unable to definitively locate the foreign body due to left breast swelling and contusions. The patient was therefore advised to allow for the swelling to decrease before undergoing evaluation for surgical extraction.

**Figure 3:**
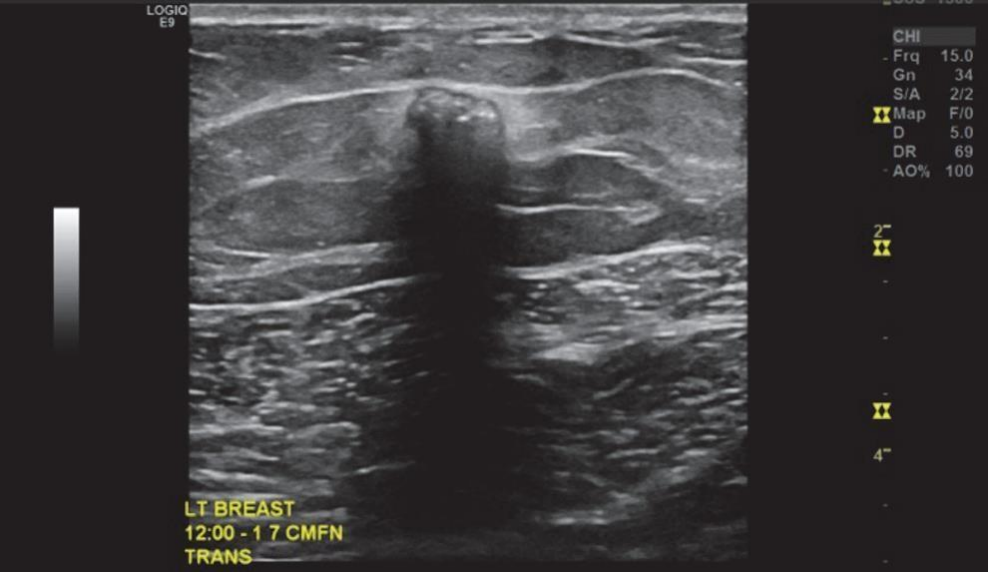
Left breast ultrasound.

Two months later, the patient had continued pain and was referred to breast surgery. A left breast ultrasound (Figure 3) revealed a 0.9 cm × 3.4 cm linear hypoechoic foreign body with features consistent with a retained metallic object. The patient reported daily intermittent pain (7/10 at its worst) associated with the puncture wound and retained object. Physical examination of the left breast did not demonstrate any palpable masses, deformities, or tenderness at the expected site of the foreign body. The areolar puncture wound was now well healed, and there were no signs of infection. Surgical excision was recommended as the patient continued to experience pain associated with the foreign body.

**Figure 4:**
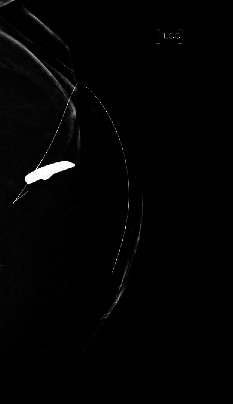
Preoperative wire localization of the foreign body.

**Figure 5:**
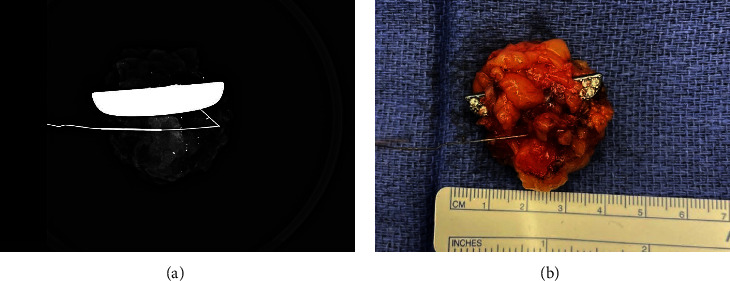
Intraoperative specimen mammogram (A) and specimen (B) demonstrating the retained foreign body and intact wire.

The patient underwent subsequent excision of the foreign body under general anesthesia. Radiology assisted with an ultrasound-guided wire localization of the foreign body given the unclear location of the object on physical exam and her large body habitus (Figure 4). Intraoperatively, the foreign body was noted to have migrated away from the puncture wound and was now deep within the breast tissue. Specimen mammography (Figure 5A) confirmed localization and excision of the metallic object and that the guide wire was removed intact. On gross examination (Figure 5B), a metallic object with rhinestones was noted, which the patient later confirmed was a decorative emblem decal she had on her steering wheel. The postoperative course was uncomplicated, and the patient had resolution of left breast pain.

In this case report, the outcome measured was the patient's pain levels, which were assessed using the numerical rating scale (NRS) [12]. At the time of presentation to the emergency department, the patient reported a pain level of 8/10. During the follow-up evaluation 1 week later, the patient reported intermittent pain that persisted over the following 2 months at 7/10 at its worst until the time of surgery. At the final postoperative evaluation, the patient reported complete resolution of pain, with a pain level of 0/10.

## 3. Discussion

Wire localization and image guidance enable precise localization of the foreign body, enhancing excisional accuracy and reducing additional trauma to the breast tissue. However, there exists a minimal risk of guide wire breakage and subsequent retention as a foreign body [3, 13]. Intraoperative specimen mammogram can alert surgeons in real time of retained wire fragments. However, it is noted that the retained wire fragments are at low risk for migration. In a series of 19 reported cases, 12 retained wire fragments were noted to be stable at 96 months later with no migration [14].

While foreign bodies in the breast can persist without intervention, surgical excision is warranted if patients exhibit associated symptoms or if there is an increased risk of complications, such as abscess or granuloma formation [3, 10]. Techniques for excision of a nonpalpable object in the breast include guide wire localization, fluoroscopy, mammotome biopsy, and radioguided occult lesion localization [3]. Guide wire was chosen for this patient due to the cost-effective and routine nature of the technique. Additionally, wire localization was favorable in this case of a large breast as wireless localization devices have signal limitations at farther distances. At our institution, the SAVI wireless localization device is used, and the manufacturer's guidance is placement for lesions up to 5 cm deep [15].

The foreign body impaled the breast during the patient's motor vehicle accident, facilitated by the angle and force of impact, and likely amplified by the deployed airbag. The patient's large, dense breasts may have mitigated the trajectory of the object and contributed to the nonpalpable nature of the object during physical examination and overall stable ED course. In a large study of over 3000 breast trauma patients with open wounds, those classified as punctures or lacerations had a higher rate of operative intervention than those with abrasions or contusions. The operative interventions were for all causes and were associated with a higher overall injury severity score and not related to the breast trauma itself [11]. This has led to a proposed tiered algorithm for treatment of breast injuries, where simple breast injuries, defined as an abrasion, small laceration, or pain over the affected breast, were managed conservatively [16]. As in our case, our patient was stable with no other injuries found on workup and was initially discharged.

Notably, the National Highway Traffic Safety Administration recently advised drivers against placing decorative emblem decals on their steering wheels as these may become projectile objects in the event of a motor vehicle accident [10]. While the patient's outcome was favorable, it highlights safety precautions that should be taken regarding the addition of this type of accessory. Additionally, awareness of breast trauma is important as it is often overlooked.

## Data Availability

No data available for this case report.

## References

[1] Steelman V. M., Shaw C., Shine L., Hardy-Fairbanks A. J. (2019). Unintentionally Retained Foreign Objects: A Descriptive Study of 308 Sentinel Events and Contributing Factors. *The Joint Commission Journal on Quality and Patient Safety*.

[2] Franco C., Moskovitz A., Weinstein I., Kwartin S., Wolf Y. (2021). Long Term Rigid Retained Foreign Object After Breast Augmentation: A Case Report and Literature Review. *Frontiers in Surgery*.

[3] Dal F., Okmen H., Kucuk Yilmaz M., Sari S., Nazli M. A., Arslan E. (2017). Extraction of a Foreign Body from the Breast Using Radio-Guided Occult Lesion Localization (ROLL): Metallic Foreign Body in the Breast. *European Journal of Breast Health*.

[4] Akyol C., Çakmak A., Kepenekci I., Erkek A. B., Baskan S. (2008). Metallic Foreign Body in the Breast. *Journal of Breast Health*.

[5] Monib S., Anis K. (2020). Iatrogenic Breast Foreign Body Seen on a Screening Mammogram. *Indian Journal of Surgery*.

[6] GTshimbidi G. (2022). Bizarre Foreign Body of the Breast Secondary to Gender-Based Violence. *South African Journal of Surgery*.

[7] Laine H. R., Kurunmäki H., Koskimies A. I. (2008). Intraductal Foreign Body in the Breast Found on Sonography. *Journal of Ultrasound in Medicine*.

[8] NHTSA’s National Center for Statistics and Analysis (2022). Overview of Motor Vehicle Traffic Crashes in. https://crashstats.nhtsa.dot.gov/Api/Public/ViewPublication/813560.

[9] Nguyen B. P., Khouri A. J., Perez-Rodriguez F., Kirkpatrick V. E., Cassaro S. (2024). Steering Wheel Aftermarket Rhinestone Emblem Projectile Injury in a Motor Vehicle Collision: A Case Report. *Cureus*.

[10] NHTSA Consumer Alert: Don’t Buy or Use Steering Wheel Decorative Emblem Decals. https://www.nhtsa.gov/press-releases/consumer-alert-steering-wheel-decorative-emblem-decals.

[11] Hager M., Spencer A., Wegener A., Lee H., Fillion M., Yon J. (2023). Breast Trauma: A United States-Based Epidemiological Study From 2016 to 2019. *Cureus*.

[12] Williamson A., Hoggart B. (2005). Pain: A Review of Three Commonly Used Pain Rating Scales. *Journal of Clinical Nursing*.

[13] Montrey J. S., Levy J. A., Brenner R. J. (1996). Wire Fragments after Needle Localization. *American Journal of Roentgenology*.

[14] Martaindale S., Scoggins M., Bassett R. L., Whitman G. (2022). Retained Localization Wire Fragments in the Breast: Long-Term Follow-up. *Current Problems in Diagnostic Radiology*.

[15] Falcon S., Weinfurtner R. J., Mooney B., Niell B. L. (2018). SAVI SCOUT® Localization of Breast Lesions as a Practical Alternative to Wires: Outcomes and Suggestions for Trouble-Shooting. *Clinical Imaging*.

[16] Sanders C., Cipolla J., Stehly C., Hoey B. (2011). Blunt Breast Trauma: Is There a Standard of Care?. *The American Surgeon*.

